# Construction of Infectious cDNA Clone of Brassica Yellows Virus Isolated from Strawberry and Establishment of TaqMan RT-qPCR

**DOI:** 10.3390/plants11233380

**Published:** 2022-12-05

**Authors:** Xiaoli Zhao, Chengyong He, Dehang Gao, Tengfei Xu, Xiaofeng Li, Junjie Liu, Shifang Li, Hongqing Wang

**Affiliations:** 1 Department of Fruit Science, College of Horticulture, China Agricultural University, Beijing 100193, China; 2 State Key Laboratory for Biology of Plant Diseases and Insect Pests, Institute of Plant Protection, Chinese Academy of Agricultural Sciences, Beijing 100193, China

**Keywords:** strawberry virus, brassica yellows virus, complete genome sequence, infectious clone, TaqMan RT-qPCR

## Abstract

The natural host range for brassica yellows virus (BrYV) is generally limited to Cruciferae. However, we found that BrYV can naturally infect strawberry. The full-length genome sequences of BrYV-MB (accession No. MZ666129) and BrYV-HY (accession No. ON060762) identified in strawberry from Yantai and Beijing, China, were obtained by high-throughput sequencing (HTS) combined with the RT-PCR and RACE techniques. The complete genome sequences of BrYV-MB and BrYV-HY are 5666 nt and contain six open reading frames (ORFs). The two isolates have the highest nucleotide (nt) sequence identity of 99.0%. The infectious cDNA clone of BrYV-HY was constructed through homologous recombination and used to agroinfiltrate *Nicotiana benthamiana* and *Arabidopsis thaliana*. The inoculated leaves of *N. benthamiana* showed necrotic symptoms after 4 days of inoculation (dpi), and the systematic leaves of *A*. *thaliana* exhibited purple symptoms at 14 dpi. To develop a rapid and high-sensitive method for the detection of BrYV, a TaqMan real-time fluorescence quantitative RT-PCR method (TaqMan RT-qPCR) was established. Under optimum reaction conditions, the sensitivity of the detection was as low as 100 fg and approximately 100-fold more sensitive than the conventional RT-PCR, so it can be used in large-scale testing.

## 1. Introduction

Strawberry (*Fragaria × ananassa* Duch.) is one of the most important horticultural plants grown in many areas of the world. In 2020, China was the largest strawberry producer, with an estimated 3.32 million tons produced, accounting for approximately 37.5% of total crop production (FAOSTAT, http://faostat.fao.org, accessed on 1 March 2022). However, strawberry yield and quality have been impacted by the occurrence and severity of viral diseases [1]. With the rapid development of high-throughput sequencing (HTS) technology in recent years, many viruses, including strawberry polerovirus 1 (SPV1), cucumber mosaic virus (CMV), tobacco streak virus (TSV), strawberry-associated virus 1 (SaV1), strawberry virus 1 (StrV-1), and others, have been reported to naturally infect strawberry plants [2,3,4,5,6]. Currently, approximately 30 viruses and 1 viroid have been reported to infect strawberry [7]. Recently, we found that strawberry is a natural host of brassica yellows virus (BrYV) in China for the first time [8]. BrYV is widespread in China, Japan, and South Korea, and it primarily infects Cruciferous crops such as cabbage, rappini, and radish, causing leaf yellowing, curling, and necrosis in the field [9,10,11]. BrYV is a phloem-restricted virus; it may enter the host phloem by aphid transmission and *Agrobacterium* infiltration, but it cannot be transferred mechanically [8,12].

Brassica yellows virus, a member of the genus *Polerovirus* in the family *Solemoviridae*, has a spherical virion with a positive-sense single-stranded RNA genome [9]. To date, three distinct genotypes, BrYV-A, BrYV-B, and BrYV-C, have been identified [9,10]. The genome of BrYV is approximately 5.7 kb in length and contains seven open reading frames (ORFs), a short 5′ untranslated region (UTR), an intergenic non-coding region (IR) between ORF2 and ORF3, and a 3′-UTR without tRNA-like structure or poly (A) tail [13]. ORF0 encodes the P0 protein, which acts as a gene-silencing suppressor; ORF1 encodes the P1 protein, which contains protease, helicase, and VPg motifs; ORF2 produces a P1-P2 fusion protein related to BrYV replication; ORF3a encodes a 3a protein related to viral system movement; ORF3 encodes the coat protein (CP); ORF4 translates the viral movement protein; ORF5 encodes an RTP protein by the read-through strategy from ORF3, associated with aphid transmission and phloem limitations [13].

Previously, we found that BrYV can naturally infect strawberry in China [8], but its molecular characteristics remain largely unknown. Viral infectious cloning is the basis for RNA virus research constructed using reverse genetic technology [14]. Although a lot of viruses have been found in strawberry, only strawberry mild yellow edge virus (SMYEV) and strawberry vein banding virus (SVBV) infectious cloning have been successfully performed so far [15,16]. Therefore, the development of a full-length infectious clone of BrYV strawberry isolate allows for in-depth research on strawberry virus pathogenicity. Rapid, specific, and sensitive virus detection methods are essential for the investigation of viruses. Many methods have been used for the detection of strawberry viruses, including reverse-transcription PCR (RT-PCR), enzyme-linked immunosorbent assays (ELISA), and real-time fluorescence quantitative RT-PCR (RT-qPCR) [17,18,19]. Among these methods, conventional RT-PCR is known to be less sensitive than real-time RT-qPCR, and ELISA is restricted by the preparation of highly effective antibodies. Thus, RT-qPCR has been widely used in the detection of a variety of fruit tree viruses due to its sensitivity, accuracy, and specificity. However, there have been fewer reports on the use of RT-qPCR in the detection of strawberry viruses.

In this study, the full-length genome sequences of BrYV-MB (GenBank accession No. MZ666129) and BrYV-HY (GenBank accession No. ON060762) were obtained from strawberry, and the genome characteristics were characterized. The infectious cDNA clone of BrYV-HY was constructed, which could systemically infect *Nicotiana benthamiana* (*N*. *benthamiana*) and *Arabidopsis thaliana (A. thaliana)*. In addition, a TaqMan real-time fluorescence quantitative RT-PCR (TaqMan RT-qPCR) method was established for the rapid, sensitive, and effective detection of BrYV in strawberry, which can be used in large-scale testing.

## 2. Results

### 2.1. Genome Characteristics of BrYV Isolates Identified in Strawberry

The genome characteristics of BrYV isolates identified in strawberry have not been previously studied. To characterize the molecular features of BrYV isolated from strawberry, the complete nucleotide sequences of two BrYV isolates were cloned and sequenced. The complete genomes of BrYV-MB (GenBank accession No. MZ666129) and BrYV-HY (GenBank accession No. ON060762) are both 5666 nt in length. Sequence analysis revealed that the genomes of BrYV-MB and BrYV-HY contain six ORFs that encode six proteins, respectively. The first ORF (ORF0), beginning at nt 32, terminates at nt 781 and encodes putative P0 proteins of 29.0 kDa (BrYV-HY) and 29.1 kDa (BrYV-MB). ORF1 (nt 174–1985) encodes a 65.8 KDa P1 protein of 603 amino acid (aa) residues. ORF2 (nt 2156 to 3268) was presumed to be translated by a-1 frameshift and encoded a putative P1–P2 fusion protein of 31.6 kDa. The intergenic regions (IRs) between ORF1 and ORF2 of BrYV-HY and BrYV-MB are 203 nt. ORF3 (nt 3472 to 4080) encodes a major CP protein of 22.5 kDa. ORF4 (nt 3503 to 4030) is entirely embedded in ORF3 and encodes a movement protein (MP). The final ORF5 (nt 4267 to 5481) is directly adjacent to ORF3, with ORF3-ORF5 potentially encoding read-through proteins of 75.1 kDa beyond the stop codon of ORF3.

**Figure 1:**
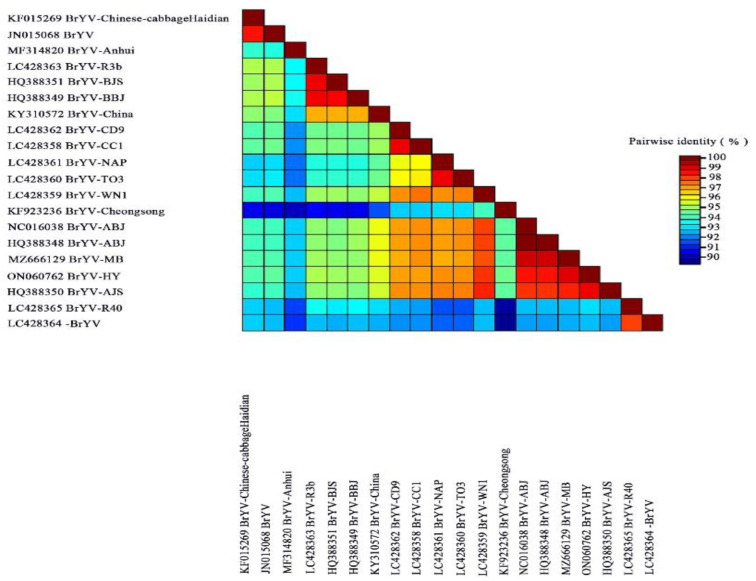
The distribution of pairwise identity scores of the complete genomic sequences of brassica yellows virus (BrYV) isolates was determined using the MUSCLE multiple sequence alignment program in SDT software. Abbreviations and GenBank accession numbers are as follows: BrYV China isolates: BrYV Chinese cabbage-Haidian (KF015269) was isolated from cabbage; BrYV (JN015068) was isolated from Raphanus sativus; BrYV-Anhui (MF314820) was isolated from tobacco; BrYV-BJS (HQ388351) and BrYV-AJS (HQ388350) were isolated from Brassica campestris L.; BrYV-BBJ (HQ388349), BrYV-ABJ (NC016038), and BrYV–ABJ (HQ388348) were isolated from Brassica napus var. napobrassica; BrYV-MB (MZ666129) and BrYV-HY (ON060762) were isolated from strawberry. BrYV Japan isolates: BrYV-R3b (LC428363), BrYV-R40 (LC428365), and BrYV (LC428364) were isolated from Raphanus sativus; BrYV–CD9 (LC428362) was isolated from Brassica oleracea var. capitate; BrYV-CC1 (LC428358) was isolated from Brassica rapa subsp. Pekinensis; BrYV–NAP (LC428361) was isolated from Brassica napus; BrYV-TO3 (LC428360) was isolated from Brassica rapa subsp. Rapa; BrYV-WN1 (LC428359) was isolated from Sinapis alba. BrYV South Korea isolate BrYV-Cheongsong (KF923236) was isolated from Chinese cabbage.

A comparison of the complete sequences of BrYV-HY and BrYV-MB revealed 99.0% nt identity. Multiple alignment showed that P0, P1, P2, CP, MP, and P5 of BrYV-HY are 96.3%, 98.8%, 99.6%, 99.5%, 99.8%, and 99.2% identical, respectively to BrYV-MB at the nucleotide level, and 95.2%, 98.3%, 100%, 100%, 99.4%, and 99.3% identical at the amino acid level. The nucleotide sequences of BrYV-HY and BrYV-MB were compared to those of other BrYV isolates. The entire genome sequence comparison showed that all 20 BrYV isolates share relatively high sequence homology (89.0–99.0%) with each other. Among them, BrYV-HY, BrYV-MB, and BrYV-ABJ share very high nucleotide sequence identities (99.0%) with each other, and a 92.1%–98.4% sequence identity with other BrYV isolates (Figure 1).

**Figure 2:**
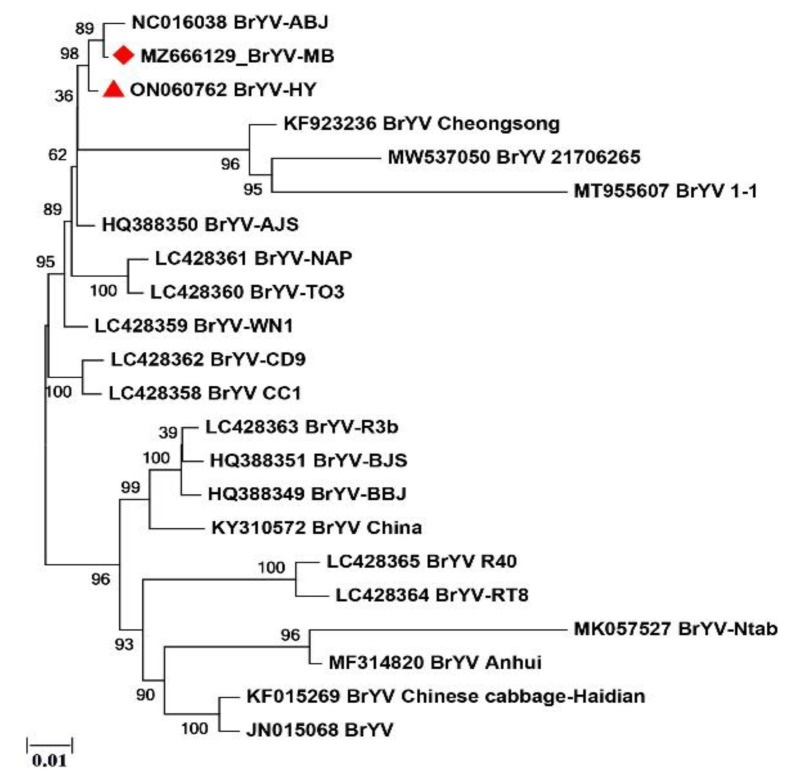
Phylogenetic relationship of the BrYV strawberry isolates with other isolates from brassica. The phylogenetic tree was constructed by MEGA version 7 using the neighbor-joining method with 1000 bootstrap replications based on the full-length nucleotide sequences. Sequences obtained in this study are marked with the red diamond and triangle.

The phylogenetic tree was constructed based on the full-length genomic sequences of the BrYV isolates. The phylogenetic relationships between the BrYV strawberry isolates and the other isolates were analyzed. It was determined that the BrYV isolates obtained in this study had the closest genetic relationship with BrYV-ABJ (Figure 2).

### 2.2. Pathogenicity Analysis of BrYV Infectious Clone

**Figure 3:**
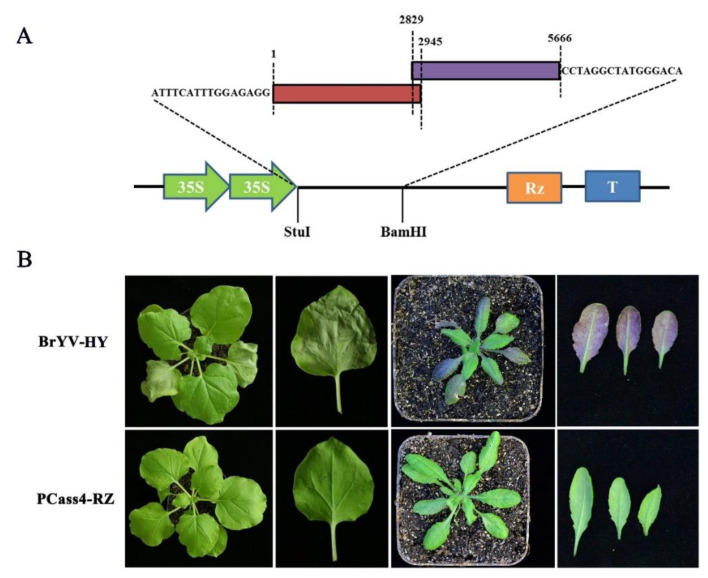
Pathogenicity analysis of BrYV-HY in *N. benthamiana and A. thaliana plants*. (**A**) Schematic representation of the construction of the full-length cDNA clone of BrYV-HY. Two overlapping segments corresponding to the complete genome of BrYV-HY were cloned into the binary expression vector pCass4-Rz between the *StuI/BamHI* sites by homologous recombination. (**B**) Symptoms of *N. benthamiana and A. thaliana* plants inoculated with BrYV-HY. Symptoms were photographed at 4 and 14 days post-inoculation (dpi). Experiments were repeated three times, and 10−15 plants were used for each inoculation.

**Figure 4:**
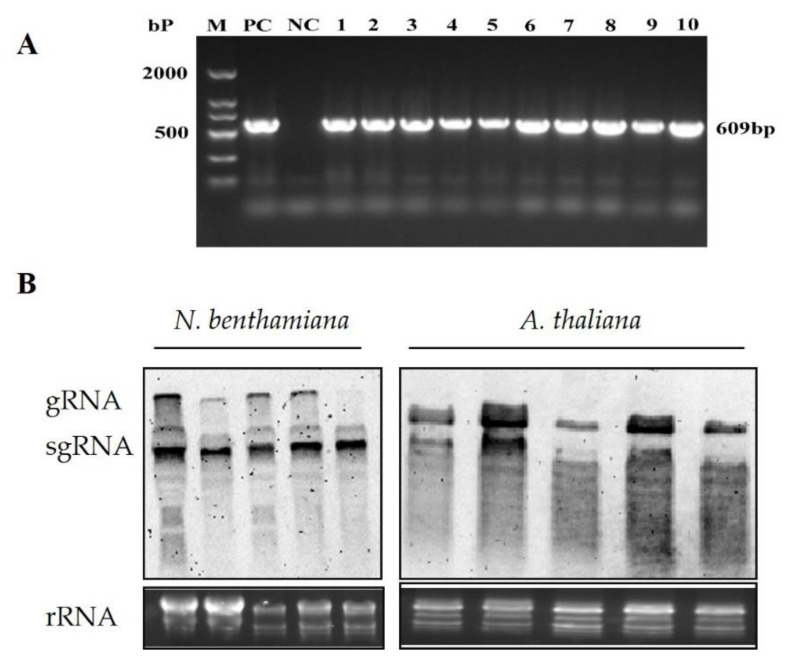
Detection results of BrYV in inoculated *N. benthamiana* and *A. thaliana* at 14 dpi. (**A**) RT-PCR for the detection of infection by BrYV-HY in the upper leaves of *N. benthamiana* (1–5) and *A. thaliana* (6–10) at 2 weeks post-inoculation with the primers BrYV-CP-F/BrYV-CP-R. PC, positive control; NC, negative control. (**B**) Northern blot analysis of BrYV accumulation in inoculated *N. benthamiana* and *A. thaliana* at 14 dpi using a DIG-labeled specific probe targeting BrYV CP coding sequences. Five independent plants were used to extract the total RNAs. The rRNAs below demonstrate the equal loading of the total RNAs.

The full-length BrYV-HY cDNA was divided into two overlapping amplified fragments using specific primers and ensuring that there was at least a 15-base-pair overlap, and then homologous recombination was used to insert the fragments into the binary expression vector pCass4-Rz between the *StuI/BamHI* sites (Figure 3A). The constructed recombination vector was transformed into EHA105 and inoculated with *N. benthamiana* and *A. thaliana*, and symptom development was monitored. The inoculated leaves of *N. benthamiana* exhibited severe necrosis symptoms at 4 dpi, while there were no obvious symptoms in the upper leaves. *A. thaliana* plants infiltrated with BrYV-HY displayed abnormal phenotypes compared to the wild-type plants, such as slowed plant growth and purple leaves at 14 dpi (Figure 3B), it was interesting that the inoculated A. thaliana leaves turned purple earlier than systemic leaves inoculated with BrYV-HY. To confirm the presence of BrYV in all infiltrated plants, the total RNA was extracted from the systemic leaves at 14 dpi and then RT-PCR was performed. Viral RNA accumulation on newly emerging leaves was tested by Northern blot analysis using digoxigenin (DIG)-labeled probes targeting the CP of BrYV. BrYV was detected in all inoculated *N. benthamiana* and *A. thaliana* plants (Figure 4A,B). These results indicate that the constructed BrYV-HY infectious clone had infectious activity.

### 2.3. Reaction Optimization of TaqMan RT-qPCR Assays

The reaction conditions, including the primer and probe concentrations, were optimized to ensure specific amplification. PCR efficiency, smooth amplification curves, and low Cq values were used to determine optimal doses. The primer screening concentrations were 0.2 μmol/L, 0.4 μmol/L, 0.6 μmol/L, 0.8 μmol/L, and 1 μmol/L. The probe screening concentrations were 0.1 μmol/L, 0.2 μmol/L, 0.3 μmol/L, 0.4 μmol/L, and 0.5 μmol/L. The results show that the Cq value of the RT-qPCR reaction was the lowest, and the fluorescence signal intensity increased the most when the primer concentration was 0.2 μmol/L and the probe concentration was 0.1 μmol/L (Table 1). Thus, the optimal combination of the primer and probe concentrations for TaqMan RT-qPCR amplification was selected in the following analysis.

### 2.4. Validation of the TaqMan RT-qPCR Assay

**Figure 5:**
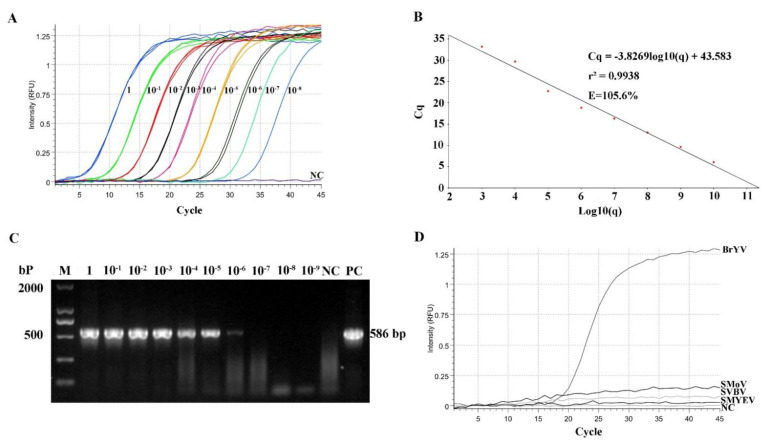
RT-qPCR detection of BrYV. (**A**) The sensitivity of the RT-qPCR. Ten-fold dilutions of the standard plasmid of BrYV were used as templates, starting from 1 μg/μL (1). RNase-free H_2_O was used as a negative control (NC). (**B**) Standard curves obtained for the BrYV quantification. The horizontal axis reports a series of ten-fold dilutions of the recombinant plasmid, while the vertical axis shows the Cq values obtained in the reactions. The R^2^ was used to evaluate the accuracy of the results obtained in triplicate assays (the closer it is to 1, the better). RT-qPCR was performed in three replicates. (**C**) Comparison of the sensitivity of RT-qPCR and RT-PCR. Ten-fold dilutions of the recombinant plasmid of BrYV were used as templates, starting from 1 μg/μL. RNase-free H_2_O was used as a negative control (NC). The prepared templates were detected simultaneously by RT-qPCR (**A**) and RT-PCR (**C**). (**D**) Evaluation of the specificity of RT-qPCR.

The recombinant plasmid was diluted 10 times continuously (a total of 10 gradients from 9.86 × 10^10^ copies/μL to 9.86 × 10^1^ copies/μL) and used as the template. The results indicate that the amplification curve of each dilution template exhibited a gradient distribution, and it was able to detect up to a 10^−8^-fold dilution of plasmid (Cq value = 33) (Figure 5A). The RT-qPCR assays exhibited good linearity of amplification with a high determination coefficient (R^2^ = 0.993) and high efficiency (E = 105.6%) (Figure 5B). In addition, the sensitivity of the RT-qPCR was verified; it was limited to a 10^−6^-fold dilution using the same samples (Figure 5C), indicating that the sensitivity of the TaqMan RT-qPCR detection system established in this study was 100 times higher than that of conventional RT-PCR. The BrYV-specific detection results indicate that only the amplification with BrYV isolates produced a standard amplification curve with a low Cq at 18, whereas no amplification was obtained with any of the other viruses or the healthy strawberry plant (Figure 5D). The results demonstrate that the established RT-qPCR detection method is highly specific for the detection of BrYV. To analyze the repeatability of the RT-qPCR, the standard plasmid, with 10^8^ copies/μL, was used as the template, and three replicates were performed each time. The results show that the reproducibility was high with an intra-assay coefficient of variation lower than 0.45% and an inter-assay coefficient of variation was 0.27% (Table 2), indicating that the established TaqMan RT-qPCR method has excellent repeatability and good detection stability.

## 3. Discussion

Strawberry is a valuable economic crop that is widely grown around the world. Strawberry viruses are causing increasingly significant harm to China’s strawberry industry as strawberry planting areas expand. Many viruses have been reported to infect strawberry plants as a result of the rapid development of HTS technology, including SPV1, CMV, TSV, and StrV-1 [2,3,4,5]. Previously, we discovered that single or mixed virus infection is common in strawberries [20]. In a previous study, BrYV was discovered for the first time in strawberry plants from Shandong province, China [8]. In the present study, the complete genome sequences of BrYV-MB and BrYV-HY were obtained, and the molecular features were characterized. Furthermore, the pathogenicity of BrYV-HY was investigated, and a TaqMan RT-qPCR method for BrYV was developed.

The Polerovirus genus virus BrYV is responsible for serious diseases and monetary losses. BrYV isolates have been sequenced in large numbers, mostly from *Brassicaceae* crops [12], but no sequence information is available on *Rosaceae* plants. BrYV was first identified in Brassica crops from China in 2011, and it is distinguished from turnip yellows virus (TuYV) based on differences in ORF0 and ORF5. Three different genotypes (BrYV-A, BrYV-B, and BryV-C) were later identified in Chinese cabbage and radish [9,10]. BrYV has a very narrow range of hosts, and generally only infects Cruciferous crops. In this study, strawberry plants with symptoms of chlorotic spots were tested by HTS, and two new BrYV isolates in strawberry were identified. The two sequences were very similar to each other at both the nucleotide and amino acid levels. The BrYV strawberry isolates obtained in this study share relatively high sequence homology (92.2–99.0%) with other isolates. Sequence alignment and phylogenetic analysis revealed that the strawberry isolates BrYV-MB and BrYV-HY share the closest genetic relationship with BrYV-A (Figure 2).

In this study, a full-length infectious cDNA clone of BrYV-HY was constructed, and its infectivity was tested by the agroinfiltration of *N. benthamiana* and *A. thaliana*. After 4 days post-inoculation, the inoculated leaves of *N. benthamiana* showed obvious necrotic symptoms, but no obvious symptoms emerged in the upper leaves. BrYV was detected in the systemic leaves by RT-PCR and Northern blot at 14 dpi, which is consistent with the inoculation symptoms of BrYV isolates from Brassica crops [12]. NbRAF2 in *N. benthamiana*’s nucleus has antiviral activity against BrYV-A infection, according to a previous study, and BrYV-A P0^BrA^ interacts with NbRAF2 and changes its localization pattern to promote virus infection [21]. *A. thaliana* inoculated with BrYV-HY showed purple leaf symptoms in leaves within one month of inoculation, with a very high infection rate of 100%. The MP protein of BrYV upregulates anthocyanin accumulation, causing purple leaf symptoms to appear on *Arabidopsis thaliana* [13]. In this study, we also tested the BrYV infectious clone in strawberry through agroinfiltration. However, the inoculated strawberry seedlings were negative for BrYV, indicating the difficulty of inoculating *Rosaceae* plants. This may be because the leaves of strawberry are thicker and it is more difficult for the virus particles to enter the leaf cells. Further attempts need to be made to fulfill Koch’s postulates on strawberry. In general, the construction of the infectious full-length cDNA of BrYV provides a basic tool for studying the functional genomics and molecular mechanism of BrYV replication, pathogenicity, and host interaction.

Disease control relies on accurate viral identification in strawberry production. Common methods for detecting strawberry viruses include ELISA, RT-PCR, and RT-qPCR [17,19,22]. In previous studies, multiplex RT-PCR methods have been developed to detect three genotypes of BrYV simultaneously [23]. Poleroviruses are phloem-limited and produce low titers in infected plants [9]; therefore, it is very important to establish a highly sensitive detection method. This study is the first report on the use of developing a TaqMan RT-qPCR assay for the detection of BrYV. The approach is very specific for BrYV detection, and other frequent strawberry viruses, such as SMoV, SVBV, and SMYEV, do not interfere with BrYV detection. In addition, the sensitivity of the RT-qPCR assay described in this study is about 100 times higher than that of conventional RT-PCR. The RT-qPCR assay demonstrated in this work may be employed in large-scale testing for BrYV due to its benefits of high specificity, sensitivity, repeatability, and much faster speed when compared to traditional RT-PCR.

## 4. Materials and Methods

### 4.1. Plant Materials

*N. benthamiana* and *A. thaliana* grown in soil were maintained in a greenhouse at 25 °C with humidity of 70%, and under a 16 h light/8 h dark cycle.

### 4.2. Amplification of BrYV Isolates

Strawberry leaves with symptoms of chlorotic spots (Figure S1) were collected from Yantai and Beijing, China, in August 2020. A total of 89 samples were quickly frozen in liquid nitrogen and stored in a −80 °C refrigerator. Then, 8 strawberry leaves (Hokowase: 2, Mibao: 2, Sagahonoka: 2, Monterey: 2) with typical viral symptoms were pooled to construct a cDNA library for HTS. RNA-Seq and bioinformatics analysis were performed as described previously [8]. Based on the assembled contigs and the complete nucleotide sequences of BrYV in GenBank, three primer pairs (BrYV-gap1-F/BrYV-gap1-R, BrYV-gap2-F/BrYV-gap2-R, and BrYV-gap3-F/BrYV-gap3-R) were designed for amplifying three bridged segments corresponding to the complete genomes of BrYV-HY and BrYV-MB, respectively. The 5′- and 3′-terminal sequences were determined using a rapid amplification of cDNA ends (RACE) 5′/3′ kit (Clontech, Mountain View, CA, USA) following the manufacturer’s instruction. ORFs were predicted by ORF Finder and SanpGene. The phylogenetic tree was constructed based on the full-length genomic sequences of the BrYV using the neighbor-joining method in MEGA version 7 software with 1000 bootstrap replicates.

The first-strand complementary DNA (cDNA) was synthesized by reverse transcription using M-MLV reverse transcriptase (Promega, Beijing, China) in a 10 μL reaction mixture containing 1 μg of total RNA, 2 μL of 5× M-MLV buffer, 1 μL of dNTP Mix (2.5 mM), 1 μL of random primer (10 μM), and 0.25 μL of RNase inhibitor. After mixing well, the mixture was centrifuged instantaneously, incubated at 37 °C for 1 h, and then stored at −20 °C for later use. RT-PCR was performed using 25 μL of the reaction mix, including 12.5 μL of 2× TransStar FastPfu PCR SuperMix, 1 μL each of two sets of primers (10 μM), and 2 μL of sample cDNA. The reaction was carried out under the following conditions: pre-degeneration at 95 °C for 5 min, followed by 35 cycles at 95 °C for 30 s, Tm for 30 s, 72 °C for 1 kb/min, and a final extension at 72 °C for 5 min. The PCR products were purified with an AxyPrep DNA Gel Extraction Kit (Corning, New York, NY, USA) and then cloned into pTopo Blunt vector (Aidlab, Beijing, China). The conjugate products were transformed into *E. coli* DH5α, and the single clones were sequenced by Sangon Biotech (Shanghai, China) Co., Ltd. All primers used in this study are listed in Supplementary Table S1.

### 4.3. Construction of Infectious Full-Length cDNA Clone of BrYV

Based on the assembled contigs, two pairs of primers (czBrYV-F/czBrYV2845-R and czBrYV2829-F/czBrYV-R), targeting 1–2845 nt and 2819–5666 nt, respectively, were designed for amplifying two overlapping segments corresponding to the complete genome of BrYV-HY. The resulting products were cloned into the binary expression vector pCass4-Rz between the *StuI*/*BamHI* sites by homologous recombination using a Trelief SoSoo Cloning Kit (TsingKe, Beijing, China). The recombinant product was transformed into *E. coli* DH5a competent cells, plated on LB medium with 100 microg/mL kanamycin, and cultured overnight at 37 °C for 16 h. Positive clones were randomly selected and the recombinant plasmid was identified by PCR and sequenced by Sangon Biotech (Shanghai, China) Co., Ltd.

### 4.4. Agrobacterium-Mediated Transformation

To test the infectivity of BrYV-HY, the recombinant plasmid was transformed into Agrobacterium tumefaciens EHA105. Colonies were grown on LB plates supplemented with kanamycin (100 microg/mL) and rifampicin (50 microg/mL) at 28 °C for 48 h. Positive colonies were re-cultured in liquid medium overnight, and then the agrobacterium cells collected from the fresh liquid medium were diluted to an OD_600_ of approximately 1 in infiltration buffer (10 mM MES, 10 mM MgCl_2_, and 150 μM acetosyringone). The resuspended cultures were incubated in darkness for 3 h prior to infiltration in 4–5 week-old *N. benthamiana* and *A. thaliana*. The inoculation experiment was repeated more than three times.

### 4.5. Northern Blot Assays

The total RNA was extracted from upper leaves collected from Agrobacterium-infiltrated plants using Transzol reagent (Transgen, Beijing, China) following the manufacturer’s instructions. Then, 3 µg of the total RNA was separated on 1.5% formaldehyde-agarose gels followed by transfer to Hybond N+ nylon membranes (GE Healthcare, Buckinghamshire, UK), and immobilization by UV cross-linking. Northern blot hybridization was performed at 68 °C overnight with a BrYV-specific CP probe, which was synthesized with a DIG RNA Labeling Kit (SP6/T7) (Roche, Switzerland), and detected using a Molecular Imager ChemiDoc XRS Imaging System (Bio-Rad, Hercules, CA, USA).

### 4.6. TaqMan Primers and Probe Design

All the available BrYV sequences from the National Center for Biotechnology Information database and the full-length sequences of BrYV-HY/MB were aligned. The highly conserved regions were further used for designing the primers and probe using the Primer3Plus website software (https://www.primer3plus.com/index.html, accessed on 1 July 2021.). The probe was labeled with the Hexachloro fluorescein (HEX) on the 5′ end, and with Black Hole Dark Quencher 2 (BHQ-2) on the 3′ end (Supplementary Table S1). The primers and probe were synthesized by Tsingke Biotechnology Co., Ltd, Beijing, China.

### 4.7. Standard Curve Preparation

RT-qPCR was performed with successive dilutions of a standard plasmid to construct a standard curve for absolutely quantifying BrYV. To construct the standard plasmid, BrYV was amplified with primers of czBrYV2829-F/czBrYV-R. The amplified products were purified using an Axy Prep DNA Gel Extraction Kit (Axygen, Union City, CA, USA) and cloned into the pTopo-Blunt vector (Aidlab, Beijing, China) following the manufacturers’ protocol. The conjugate product was transformed into *E. coli* DH5α to construct the recombinant plasmid pTopo:BrYV-R. The recombinant plasmid was diluted 10 times continuously (a total of 10 gradients from 9.86 × 10^10^ copies/μL to 9.86 × 10^1^ copies/μL) and used as positive standard templates, namely 10^−^^1^,10^−^^2^,..., 10^−^^9^. RNase-free H_2_O was used as a negative control, and each concentration was repeated three times. RT-qPCR assays were carried out on the MyGo Pro real-time PCR instrument (IT-IS Life Science Ltd., Eastleigh, UK) with a 20 μL of PCR mixture, which consisted of 0.5 μL of plasmid, 10 μL of 2× PerfectStartTM II Probe qPCR Super Mix Kit (TransGen Biotech, Beijing, China), and 0.5 μL of each primer. RT-qPCR was performed with the following thermal program: 94 °C for 30 s, 94 °C for 5 s, followed by 45 cycles at 60 °C for 30 s. The standard curve was created by plotting threshold cycle (Cq) values versus the logarithm of the template copy number over three replicates per dilution.

### 4.8. Sensitivity, Repeatability, and Specificity

To evaluate and compare the sensitivity of the established TaqMan RT-qPCR assay with the conventional RT-PCR, 10-fold serial dilutions (from 10^−^^1^ to 10^−^^9^) of the standard plasmid were prepared as templates and used in both the RT-qPCR and the conventional RT-PCR. The primers used for the RT-PCR were BrYV4395-F/BrYV4981-R. At each concentration, three technical replicates were prepared. The specificity of the RT-qPCR assay was assessed using a known positive sample of BrYV. The cDNA samples with positive SMoV, SVBV, and SMYEV were used for the RT-qPCR-specific detection of BrYV, and healthy strawberry plant was used as a negative control. The standard plasmid with a concentration of 10^8^ copys/μL was selected to test the repeatability. The Cq value, standard deviation (SD), and coefficient of variation (CV) were calculated.

## 5. Conclusions

This study determined the full-length genome sequences of BrYV isolates BrYV-MB and BrYV-HY identified in strawberry. Furthermore, an infectious cDNA clone of BrYV-HY was constructed, which systemically infected *N. benthamiana* and *A. thaliana* and caused obvious symptoms. In addition, this is the first report to establish a TaqMan RT-qPCR method for the rapid, sensitive, and effective detection of BrYV in strawberry, which can be used in large-scale testing.

## Figures and Tables

**Table 1:** The Cq value of BrYV in the TaqMan RT-qPCR assay using different primer and probe concentrations.

Primer Concentration		Probe Concentration (μmol/L)			
(μmol/L)	0.1	0.2	0.3	0.4	0.5
0.2	8.972	9.735	10.054	10.545	10.702
0.4	9.439	9.689	10.122	10.617	10.819
0.6	9.495	9.919	10.226	10.660	10.652
0.8	9.461	9.907	10.262	10.767	10.776
1.0	9.381	9.885	10.485	10.769	10.063

**Table 2:** Repeatability analysis of the established TaqMan RT-qPCR method for BrYV detection.

Cq Value			Intragroup		Intergroup	
			Mean ± SD ^a^	CV ^b^(%)	Mean ± SD	CV (%)
13.1133	13.0825	13.0940	13.09666 ± 0.016	0.12%		
13.1517	13.0474	13.1471	13.11543 ± 0.059	0.45%	13.11438 ± 0.035	0.27%
13.1502	13.1210	13.1218	13.13105 ± 0.058	0.44%		

^a^ showed mean ± standard deviation, three replicates were performed; ^b^ coefficient of variation.

## Data Availability

Not applicable.

## Supplementary Materials

The following supporting information can be downloaded at: https://www.mdpi.com/article/10.3390/plants11233380/s1, Table S1: Primers and TaqMan probe used for BrYV detection in RT-PCR and real-time RT-PCR. Figure S1: Photos of BrYV-infected strawberry plants.
