# Supplementary material for: Construction of Infectious cDNA Clone of Brassica Yellows Virus Isolated from Strawberry and Establishment of TaqMan RT-qPCR
Source: Plants (Basel). 2022 Dec 5;11(23):3380. doi: 10.3390/plants11233380 (PMC9735513; doi:10.3390/plants11233380)
Supplement: Supplementary file 1 [file plants-11-03380-s001.zip › plants-1877590-supplementary.pdf]

Supplementary Table S1. Primers and TaqMan probe used for BrYV detection in RT-PCR and real-time RT-PCR

Primers	Sequences (5' -3')	Position
**BrYV amplification**		
BrYV-gap1-F	ACGTGCCAGGAACCAAGAC	354-372
BrYV-gap1-R	CATTTCGGTGTAGACCGAAGAGC	750-772
BrYV-gap2-F	ATCGGGATCCATCCCGCATTG	1910-1930
BrYV-gap2-R	TCCGGATCAGCTCCTCAGGT	2341-2360
BrYV-gap3-F	ATGGTACAATCTCCTTTCACG	4595-4615
BrYV-gap3-R	ATTCCTTGTTGTCCTCAGAAC	4391-4951
5'RACE	GCGAGCTCCACAAGCGTGTGAGACC	543-567
3'RACE	AGGAGGTTCGCTTGCTGGCGGCAG	5199-5222
**Homologous reorganization**		
czBrYV-F	ATTTCATTTGGAGAGGACAAAAGAAACCAGGAGGGAATCC	1-24
czBrYV2845-R	CGGATGGGTTTGGGCTAAAAGGGTACCATCGCTCAG	2810-2845
czBrYV2829-F	TAGCCCAAACCCATCCGGGCGTTCAGAAAAGTGGG	2829-2863
czBrYV-R	ACAGGGTATCGGATCCACACCGAAGTGCCGTAGGG	5648-5666
**RT-qPCR**		
BrYVqp-F	GCCTCCGGTACATTGAGAATGA	4241-4262
BrYVqp-R	CCTTGTTAGGGTCGGTGGT	4402-4420
BrYV4395-F	AGCAAGTACCACCGACCCTA	4395-4414
BrYV4981-R	GAAACATTCGAGTCCCGGGT	4962-4981
BrYVq pro	HEX-ACATGAACTGGACCAACGTGGACGCCC- BHQ-2	4265-4291
**Virus Detection**		
BrYV-CP-F	ATGAATACGGTCGTGGGTAG	3472-3491
BrYV-CP-R	CTATTTGGGATTATGGAATTGGC	4058-4080

Note: The underlined sequences indicate the regions of the recombination sites

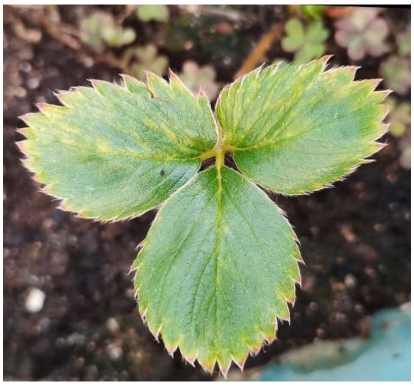

Figure S1: The photos of BrYV infected strawberry plants.
